# Dispersal of *Aphanoascus keratinophilus* by the rook *Corvus frugilegus* during breeding in East Poland

**DOI:** 10.1038/s41598-022-06227-2

**Published:** 2022-02-08

**Authors:** Ignacy Kitowski, Teresa Korniłłowicz-Kowalska, Justyna Bohacz, Anita Ciesielska

**Affiliations:** 1 Department of Zoology and Animal Ecology, University of Life Sciences in Lublin, Akademicka 13, 20-950 Lublin, Poland; 2 Laboratory of Mycology, Department of Environmental Microbiology, Faculty of Agrobioengineering, University of Life Sciences in Lublin, Leszczyńskiego 7, 20-069 Lublin, Poland; 3 Department of Molecular Microbiology, Faculty of Biology and Environmental Protection, University of Łódź, Banacha 12/16, 90-237 Łódź, Poland

**Keywords:** Ecological epidemiology, Environmental microbiology, PCR-based techniques

## Abstract

The process of dispersal of the potentially disease-causing, geophilic and keratinolytic fungal strain *Aphanoascus keratinophilus* (the perfect, sexual stage of *Chrysosporium keratinophilum*) by the rook *Corvus frugilegus* was studied. The source of *A. keratinophilus* strains was pellets of the rook, thus far not considered a carrier of this particular opportunistic pathogen. Pellets collected from breeding colonies of rooks were analysed in terms of the occurrence of keratinolytic fungi with the application of the native keratin bait method. Among the 83 rook pellets analysed, 24 (29%) were infected by keratinophilic fungi. Pure cultures of the fungi were identified to species based on traditional morphological features. Traditional mycological identification was verified by the PCR–RFLP molecular identification method as well as DNA sequencing. The obtained results showed the presence of 90 *Aphanoascus keratinophilus* strains, 6 *Chrysosporium tropicum* strains, and 3 *Chrysosporium pannicola* strains. The PCR melting profile (PCR-MP) method was used to identify intraspecies variations of the 90 analysed *A. keratinophilus* strains. The dispersal of genotypes and possible pathways of *A. keratinophilus* dispersal and infection via rook pellets were analysed.

## Introduction

Birds are considered an important carrier of pathogenic and potentially pathogenic microorganisms, including fungi^1–5^. The role of synanthropic free-living bird species, including the rook *Corvus frugilegus,* in epidemiology, has been emphasized^6^. Rooks are highly social, living and interacting in large groups, although mating tends to be monogamous^7^. The food preferences of the rook vary throughout the year. However, the food consumed is almost always connected with the soil where the birds forage^7–9^. This bird species cast pellets, i.e., undigested food debris. During the hatching period, these are mainly residues of food taken by adult birds from the soil, which causes the food to be contaminated with soil microorganisms. As omnivores, the rooks often eat food found by probing the ground in search of earthworms and other invertebrates, especially insects^10^. Rook food also contains the grains of cereals and smaller amounts of seeds of other plants^11^. Rooks have numerous roles in the ecosystem, i.e., they serve as hosts for organisms such as protozoans, helminths and fungi^12,13^.

Among the mycobiota associated with birds, particular attention should be given to a group of keratinophilic fungi, such as dermatophytes, as well as the *Chrysosporium* group (conventional term). These organisms have the capacity to breakdown keratin, such as feathers, hairs, nails, horns, and claws^14–16^. Dermatophytes have been divided into three ecological groups: geophilic and zoophilic (i.e., *Trichophyton verrucosum*, *Microsporum canis*), which can spread the infection to humans, and anthropophilic dermatophytes such as *T. rubrum* and *T. interdigitale,* causing dermatophytosis in humans^17–19^. The *Chrysosporium* group comprises over 60 species (i.e., *A. keratinophilus*, *A. fulvescens*, *Ch. tropicum*)^20^, mainly soil-borne keratinolytic species^21–23^, which are saprotrophs^24^. Although considered safe, during certain developmental stages, these fungi can convert into a pathogenic form under defined environmental conditions. Among them, pathogenic strains causing skin and nail diseases and sometimes deeper infections, particularly in immunosuppressed patients, were found, which qualifies these fungi as opportunistic pathogens^25–29^. The birds themselves rarely suffer from dermatomycosis caused by dermatophytes or by fungi from the *Chrysosporium* group. However, the most frequent fungal infections of free-living birds include mycoses of the organs (deep infections), especially of the respiratory system, caused by *Aspergillus fumigatus*^3^. Within the *Chrysosporium* group, *Chrysosporium keratinophilum* is the most common cause of surface fungal infections in humans and animals^23,27,30^. In accordance with the latest nomenclature currently in use (http://www.indexfungorum.org/), the species name of the fungus is the name of the perfect, sexual stage (teleomorph) appearing in the life cycle of some fungi in which sexual spores are generated^31^. In the case of *Chrysosporium keratinophilum*, *Aphanoascus keratinophilus* is the perfect stage of this fungus, and this name will be used hereafter in the present study. The long-term monitoring of rooks in Poland allowed us to estimate their population as 300–350 thousand pairs^32^. However, both epidemiological and ecological aspects of the propagation of fungal diseases by birds, including rooks, are not fully understood^6^. Rook pellets containing undigested food debris have not been analysed in this aspect.

The first aim of the present study was to provide evidence for rooks as carriers of potentially pathogenic strains of *Aphanoascus keratinophilus* transferred from individual adult birds. An attempt was also made to estimate the dispersion of *A. keratinophilus* connected with the production of pellets. The second goal was molecular identification and differentiation of the *A. keratinophilus* strains isolated from *Corvus frugilegus* pellets using basic genetic techniques such as PCR–RFLP and PCR-MP. To our knowledge, this molecular analysis of *A. keratinophilus* populations was performed for the first time in this study.

## Results

### Strain prevalence and identification of keratinophilic fungi using traditional and molecular methods

Among the 83 rook pellets analysed, 24 were infected by keratinophilic fungi, including 17 infected by *Aphanoascus keratinophilus* (Punsola&Cano), which represented 20,5% of all analysed pellets. Based on the traditional mycological identification method, three species of keratinolytic fungi were identified: *Aphanoascus fulvescens* (23 strains), *Aphanoascus keratinophilus* (64 strains) and *Chrysosporium tropicum* (2 strains). Ten strains were classified as *Chrysosporium* sp. (Table S1).

**Figure 1:**
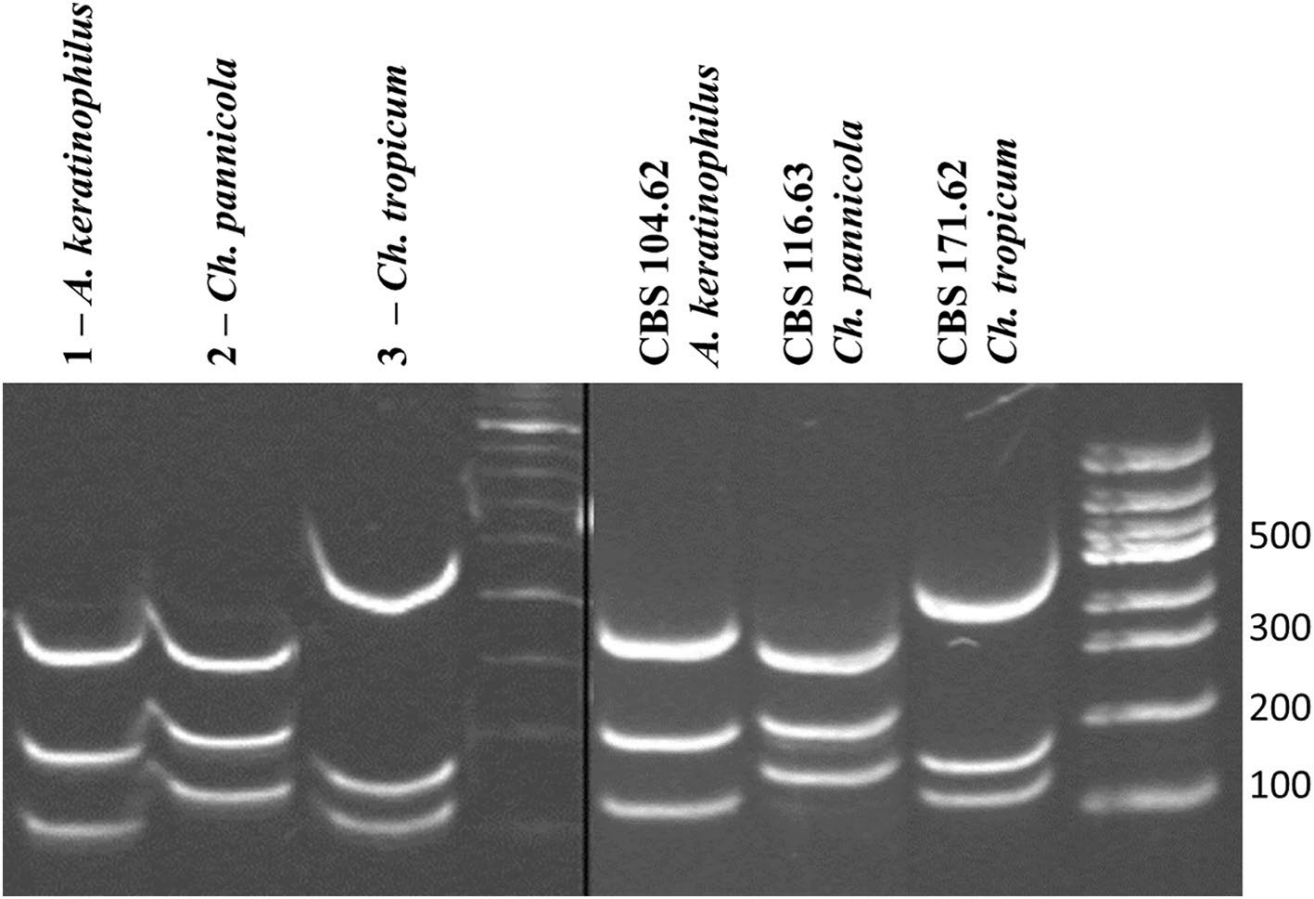
Exemplary polyacrylamide-gel electrophoresis of PCR products digested with *Hin*fI restriction enzyme. The ITS1-ITS4 set of primers was used to amplify the ITS1-5.8SrDNA-ITS2 region. Profiles A–C are characteristic of *Aphanoascus keratinophilus*, *Chrysosporium pannicola* and *Chrysosporium tropicum*, respectively. Abbreviations above lanes (1–3) correspond to the species names assigned during traditional identification. Full-length gels are shown in Supplementary Fig. S1a,b.

The genomic DNA of the reference strains (CBS 104.62; CBS 171.62; CBS 116.63) and ninety-nine collected strains of keratinophilic fungi isolated from *C. frugilegus* pellets in East Poland were amplified using universal primers ITS1 and ITS4^33^. The size of the obtained PCR products was approximately 600 bp for all strains (Fig. 1). We performed RFLP analysis for all 99 PCR products and reference strains using the *Hin*fI restriction enzyme^33,34^. Based on the DNA fragments obtained after restriction analysis, we could distinguish three patterns specific for *A. keratinophilus* (90 strains) (Punsola&Cano), *Ch. pannicola* (6 strains) (Oorschot&Stalpers) and *Ch. tropicum* (3 strains) (J.W. Carmich) (Fig. 1). In 38 cases, differences were revealed between the traditional results and those obtained using the PCR–RFLP method (Table S1). Three strains that were identified as *A. keratinophilus* using traditional methods and three strains designated *A. fulvescens* after molecular identification were *Chrysosporium pannicola* and *Chrysosporium tropicum*, respectively (Table S1). Moreover, based on traditional morphological features, twenty strains were identified as *A. fulvescens*, while after molecular identification, they were revealed to be *A. keratinophilum* (19 strains) and *Ch. pannicola* (1 strain) (Table S1). On the other hand, 2 strains identified as *Ch. tropicum* based on the PCR–RFLP method appeared to belong to *A. keratinophilus*. Collections identified by the traditional method as *Chrysosporium* sp. (10 strains) based on genetic characteristics were determined to be *A. keratinophilus* (8 strains) and *Ch. pannicola* (2 strains) (Table S1). To confirm the final results of PCR–RFLP identification, sequencing of the entire ITS1-5.8S rDNA-ITS4 DNA region of 38 misidentified strains was performed with the universal primers mentioned above. Full agreement between PCR–RFLP patterns and the sequencing results was obtained (see Supplementary material).

### PCR-MP genotyping

**Figure 2:**
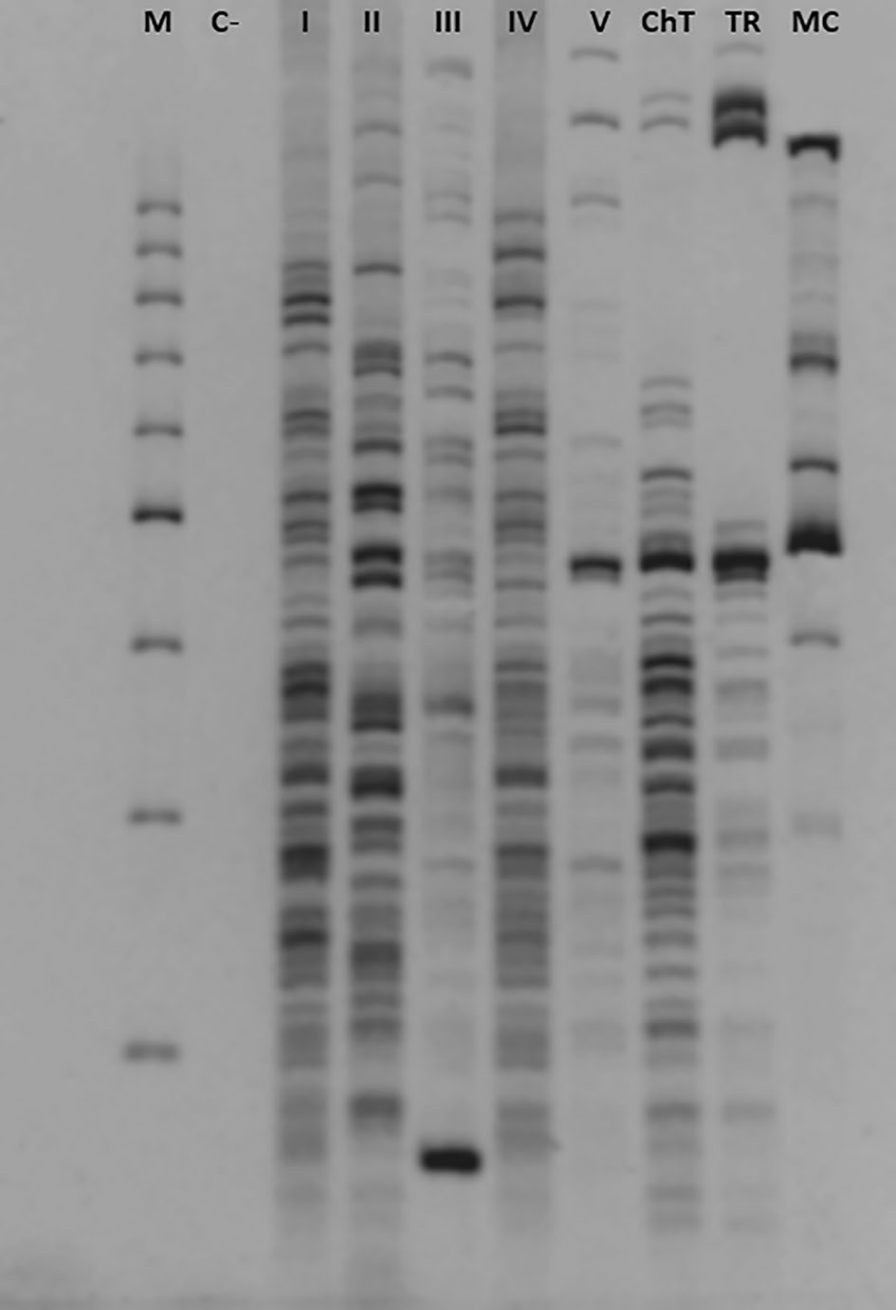
PCR-MP profiles of *A. keratinophilus* strains isolated from rook pellets using the *Bam*HI restriction enzyme. Electrophoresis of the DNA amplicons was carried out on a 6% polyacrylamide gel. The lane designated M contained the molecular mass marker (1000, 900, 800, 700, 600, 500, 400, 300, 200 bp); C(−) -negative control, lacking template DNA; positive controls designated ChT, *Chrysosporium tropicum*; TR, *Trichophyton rubrum*, MC, *Microsporum canis*.

Among 90 strains of *A. keratinophilus* originating from rook pellets, we distinguished five (I-V) PCR-MP genotypes (Fig. 2, Table 1). Genotype I was markedly predominant and represented by thirty-one, two, and three strains originating from Chelm, Wola, Uhruska, and Wierzbica, respectively. Despite the fact that genotype I was numerous in the city of Chełm, its frequency (62.0%) was statistically insignificant (χ^2^ = 2.88, df = 1, *p* = 0.08969) compared to the total frequency (38.0%) of the remaining genotypes (Table 1). Genotype II was characteristic of seven strains originating from Wola Uhruska and three localized in Siennica and Sielec. Genotype III was represented by eleven, one, and two strains originating from Chelm, Wola Uhruska, and Chojno Nowe, respectively. Genotype IV was specific for eight strains of *A. keratinophilus* originating from Chelm. The remaining nineteen strains were classified as genotype V, specific for five, three, and eleven isolates originating from Wola Uhruska, Siennica, and Chojno Nowe, respectively (Table 1). The discriminatory power of the PCR-MP method was good, yielding a Simpson's index of diversity (D) value of 0.750.

**Table 1:** PCR-MP/*Bam*HI genotypes of *A. keratinophilus* strains isolated from rook pellets and frequency of obtained genotypes.

Colony site	Total number of isolates (frequency of genotypes %)	PCR-MP/*Bam*HI genotype				
		I	II	III	IV	V
Sielec	3 (100%)	–	3 (100.0%)	–	–	–
Chojno Nowe	13 (100%)	–	–	2 (15.4%)	–	11 (84.6%)
Siennica	6 (100%)	–	3 (50.0%)	–	–	3 (50.0%)
Wola Uhruska	15 (100%)	2 (13.3%)	7 (46.7%)	1 (6.7%)	–	5 (33.3%)
Chelm	50 (100%)	31 (62.0%)	–	11 (22.0%)	8 (16.0%)	–
Wierzbica	3 (100%)	3 (100%)	–	–	–	–

The frequency of genotypes (I-V) in all 90 strains of *A. keratinophilus* originating from pellets belonging to rooks nesting in Chełm and its vicinity (Table 1) were statistically significantly different from the predicted frequency assuming an equal (20.0%) share of all genotypes (χ^2^ = 11.1796, df = 4, *p* = *0.0246*).

### Estimation of the dispersion of *Aphanoascus keratinophilus* strains using traditional and molecular methods

Based on the duration of the reproduction period (107 days) and the size of particular colonies, an estimate of *A. keratinophilus* dispersion was made (Table 2). Rooks from the studied colonies produced 2327.2 − 19761.7 pellets containing at least one *A. keratinophilus* strain during the breeding period. The geometric mean of the number of produced pellets of *A. keratinophilus* for all tested colonies amounted to at least 8250.8. Tested rooks, depending on the colony, contributed to the spread of at least 21.8–184.9 strains of *Aphanoascus keratinophilus* per day (geometric mean: at least 77.2 strains/day). Given the area of 3.14 km^2^ around the colony indicated in the methods, it was estimated that the density of strains in that area was at least 0.001–0.006 strain/100 m^2^ (Table 2) (the geometric mean for all the colonies was at least 0.003 strain/100 m^2^). The frequency of PCR-MP genotypes was also estimated for ninety strains of analysed *A. keratinophilus* (Table 1). However, detailed computations about the dispersion of PCR-MP genotypes of *A. keratinophilus* isolated from Rook’s pellets were conducted only for three colony sites where > 10 strains of analysed fungi were available (Table 3). In other cases, despite the random collection of *Corvus frugilegus* pellets, the small number of analysed strains did not guarantee the representativeness of the obtained data.

**Table 2:** Estimation of the *Aphanoascus keratinophilus* minimal strain dispersion and the minimal density of strains per 100 m^2^ area most intensively used by rooks around colonies.

Colony site	Number adult birds	Total pellet production	Pellets with at least one strain *Aphanoascus keratinophilus*	Minimal strains of *Aphanoascus keratinophilus* dispersion/day	Minimal density strains of *Aphanoascus keratinophilus*/100 m^2^
Sielec	1002	96492.6	19781.0	184.9	0.006
Chojno Nowe	792	76269.6	15635.3	146.1	0.005
Siennica	542	52194.6	10699.9	100.0	0.003
Wola Uhruska	326	31393.8	6435.7	60.1	0.002
Chelm	324	31201.2	6396.3	59.8	0.002
Wierzbica	118	11363.4	2329.5	21.8	0.001

**Table 3:** PCR-MP/*Bam*HI genotype minimal dispersion of *A. keratinophilus* strains originating from colony sites where > 10 strains of analysed fungi were available.

Colony site	Pellets with at least one strain *A. keratinophilus*	PCR-MP/*Bam*HI genotype				
		I	II	III	IV	V
Chojno Nowe	15635.3	–	–	2407.8	–	13227.5
Wola Uhruska	6435.7	855.9	3005.5	431.2	–	2143.1
Chełm	6396.2	3965.6	–	1407.2	1023.4	–
Total (N)	28467.2	4821.5	3005.5	4246.2	1023.4	15370.6
Total (%)	100	16.9	10.6	14.9	3.6	54.0

## Discussion

Currently, according to the Index Fungorum, the names and taxonomic positions of *Chrysosporium keratinophilum* are as follows: *Aphanoascus keratinophilus* Punsola&Cano; anamorph: *Chrysosporium keratinophilum* Frey ex Carmich.; *Ascomycota*, *Eurotiomycetes*, *Onygenales*, *Onygenaceae* (http://www.indexfungorum.org/names/names.asp-accesb). High, over 90%, occurrence of *A. keratinophilus* strains (at the teleomorph or anamorph stage) among keratinophilic *Chrysosporium* strains found in Rook’s pellets should be associated with the occurrence of this fungus in the soil environment and its thermal tolerance^15,35^, facilitating its survival in the digestive tract of birds. The body temperature of all free-living birds is higher than that of mammals and is approximately 42 °C^36,37^. This factor favours the survival of propagation units of *A. keratinophilus* under adverse conditions in the gastrointestinal tract of birds. This relates primarily to spores, especially teliospores, that retain vitality after passing through the gastrointestinal tract^38^. Thermal tolerance of *A. keratinophilus* strains is also an important feature in the pathogenesis of this species. This is important, especially in relation to deep infections. *A. keratinophilus* grows well at a temperature of 37 °C, which corresponds to the temperature in the human body. Deep infections caused by *Chrysosporium* strains are difficult to treat^25,39^.

The identification of *Chrysosporium* species based on phenotypic traits is difficult and can be incorrect due to the polymorphism of fungi. For that reason, the PCR–RFLP method was applied for the molecular identification of *Chrysosporium* sp. isolates on the basis of ITS1/5.8S/ITS4 region amplification and restriction analysis of the obtained PCR products. Mochizuki et al.^34^ used the *Hinf*I restriction enzyme for the successful species identification of dermatophytes by RFLP analysis of ITS regions. We decided to utilize this method as a “standard”, which allowed us to attribute the RFLP patterns obtained in our investigation to an appropriate species or genus. Traditional morphological identification, such as microscopic examination to visualize fungal elements in the sample followed by cultivation, was performed. In 38 cases, significant differences were revealed between the traditional results and those obtained using molecular methods (Table S1). To confirm the results of PCR–RFLP analysis, PCR products from randomly selected isolates were sequenced, and the nucleotide sequences were compared with the sequences collected in the NCBI database. In both cases, there was full agreement between PCR–RFLP patterns and the sequencing results (see Supplementary material). PCR–RFLP analysis revealed that the traditional identification method may frequently result in erroneous identification of keratinolytic fungi and showed that molecular methods such as PCR–RFLP can be used, at least as supplementary techniques to the traditional procedures of mycological identification.

The role of the rook *Corvus frugilegus* in the dispersion of *A. keratinophilus* in the city of Chełm was performed by our group previously^40^. We investigated the possible factors determining the significance of rookeries in the propagation of *A. keratinophilus*. We proved that the dispersion of this keratinolytic fungus is related to the size of the rook breeding population. Moreover, the presence of rook colonies in human population areas such as schools, kindergartens or hospitals may increase the epidemic risk of lung infections caused by *A. keratinophilus*^40,41^. In the present study, we demonstrated the application of the PCR melting profile as a simple method that may be used for intraspecies differentiation of analysed strains of *A. keratinophilus* isolated from pellets of *Corvus frugilegus*. This method has been successfully used for distinguishing bacterial strains such as *S. aureus* or *E. coli*^42^ and for differentiation of dermatophytes^18,43^. We chose the *Bam*HI restriction enzyme and an appropriate denaturation temperature to obtain high differentiation power with the PCR-MP method. Five types (I-V) of *A. keratinophilus* were distinguished (Fig. 2., Table 1). PCR-MP analysis proved to be a suitable technique for the detection of intraspecies genetic relatedness in *A. keratinophilus*, which will allow us to monitor the occurrence as well as distribution of these strains^18^, and demonstrated a slight similarity between the percentages of PCR-MP genotypes originating from each colony site (Table 3).

When considering the role of the rook as a carrier of *A. keratinophilus*, its food habits should be considered. The rook diet depends on the soil and contains plant material (mainly grains of cereals and smaller amounts of seeds of other plants) and ground invertebrates, especially insects and their larvae^8,9,44^, which requires actively digging and excavating the soil. This is important because *A. keratinophilus* demonstrates a high prevalence in soils from both rural and urban areas^20,45–47^, and its strains are highly prevalent in feathers of birds foraging in close relation to soil^14–16,48^. On the other hand, it was reported that^49^ individuals of *Corvus frugilegus* are able to make trips up to 40 km from their winter night communal roosts to foraging areas, which may create conditions for *A. keratinophilus* strains to be transferred long distances.

Studies carried out in the present work point to the high efficiency of the dispersion of *A. keratinophilus* strains by rooks. It should be stressed that a large accumulation of pellets occurs on shady surfaces directly under trees with breeding colonies and communal roosts^8,9^. This fact is of great epidemiological importance in densely populated urban areas. It can be assumed with a high probability that with a large production of pellets in colonies and favourable conditions for fungal growth (presence of feathers and faeces in shady areas under trees), the number of *A. keratinophilus* strains will quickly multiply. On the basis of the results obtained in our previous work^40^ and the present study, we concluded that in the long term, threats related to *A. keratinophilus* spread by rooks in urban and rural areas in Poland and Europe will decrease. Declines in the rook population, expressed by a decrease in the size of the colony, may be the reason for this phenomenon^50^. However, locally, large rook colonies located in places such as small city parks with a large number of visitors, the surroundings of hospitals, and shopping centres may pose a certain threat in the context of the dispersion of *A. keratinophilus*^40^.

**Figure 3:**
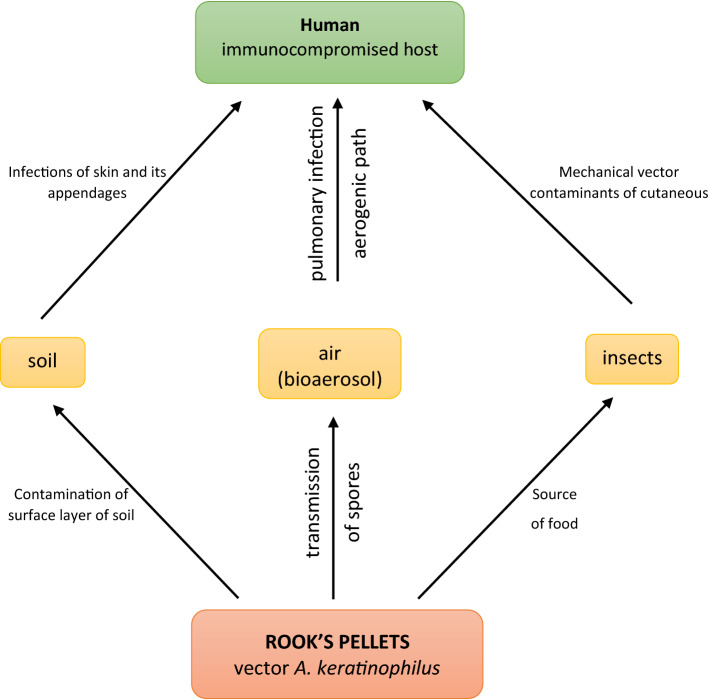
Possible pathways of dispersal and infections with opportunistic strains of *Aphanoascus keratinophilus* with the participation of rook pellets.

Thermal (“islands of heat”) and wind conditions (low average wind speeds leading to low aeration) prevailing in cities^51,52^ should also be considered significant factors contributing to the saprophytic development of *A. keratinophilus*. This species, in terms of environmental requirements, is classified as thermotolerant and hydrophilic^35^. These factors favour the dispersion of potentially pathogenic geophilic fungi carried by birds in towns and cities. In light of the results of our research and of the cited reports by other authors concerning the dispersal of *A. keratinophilus* in the environment, rook pellets can be proposed as a new link in the epidemiological chain of opportunistic pathogens. Figure 3 presents rook pellets as a source of infection of *A. keratinophilus*, considering two main pathways of transmission of that pathogen: through contaminated soil and via contaminated air, with the additional suggested possibility of dispersal of those fungi via biological vectors such as insects foraging on the pellets. It can be concluded that strains of *A. keratinophilus* isolated from pellets are more dangerous to human health than those isolated, e.g., directly from soil, as they are selected strains that have passed through the gastric-intestinal barrier and are thus more resistant to conditions prevailing in the organism, such as elevated temperature, acidic (stomach) or alkaline (intestines) conditions, and low oxido-reduction potential. It should also be mentioned that rooks are not only carriers of *A. keratinophilus* but also of other keratinophilic fungi^15^.

## Conclusion

The results of this study showed that synanthropic species, i.e., rooks, could be vectors of potentially pathogenic *A. keratinophilus* strains of soil origin and thus constitute a link in the epidemiologic chain of opportunistic fungal infections. Analysis of the species composition of *Chrysosporium* spp. isolated from rook pellets collected from breeding colonies located in East Poland demonstrates the important role of these birds in the dispersion of *A. keratinophilus* strains. These data have been reported for the first time and have no counterpart in the works of other authors because rook pellets have not been previously examined for the presence of keratinophilic fungi.

## Materials and methods

### Study area

The studies were carried out in *Corvus frugilegus* colonies localized at the following sites (Eastern Poland): Chelm (51°07′56″ N, 23°28′40″E), Chojno Nowe (51°10′44″N, 23°04′26″E), Sielec (51°02′07″N, 23°31′00″E), Siennica (51°00′22″N, 23°19′16″E), Wierzbica (51°15′44″N, 23°19′13″E) and Wola Uhruska (51°19′20″N, 23°37′30″E).

### Mycological analysis

A total of 83 rook pellets were collected in the mentioned rookeries. The pellets were collected between 20 April and 19 May 2013. To avoid potential contamination from the local soils, pellets were collected from plastic foil placed under trees with nests. The chemical composition of pellets in terms of percentage dry matter were as follows: organic C, 17.5–33; total N, 1.3–4.3; total P, 0.38–2.98; total S 0.16–0.24; Ca 1.9–12.1; Mg 0.18–0.42; K 0.10–0.28; water content 2.84–8.38%, and pH _H2O_ 7.15 (range from 6.30 to 8.02). Due to the small size of the pellets, the sample prepared for pH assays consisted of three individual pellets. The pellets collected in the field were separately analysed mycologically as single samples. After crushing, the pellets were placed on sterile Petri dishes and sprinkled with sterile chicken feathers and child hair (approximately 1 cm long) as keratin substrate^53^. Cultures were incubated at 26 °C and 37 °C for 3–4 weeks and wetted with sterile distilled water as necessary. The fungal mycelium that appeared was transferred onto Sabouraud medium with an antibiotic and actidione. The species identification of pure cultures was conducted on the basis of morphological (phenotypic) characteristics. Morphology of the colonies was analysed, including the size, structure, and colour (obverse, reverse) of the colonies and micromorphological features, including the presence of characteristic vegetative hyphae, i.e., racquet hyphae in the aerial and submerged mycelium, the presence, size, shapes and position of conidia of the aleuriospore and arthrospore types, the production and presence of chlamydospores, etc. Cultures were maintained on plates and slants (macroscopic observations) and in microcultures on agar discs (microscopic observations) on Sabouraud medium (without antibiotics) according to systematic studies^21,23,25^ and compared to the reference strains from Westerdijk Fungal Biodiversity Institute (formerly CBS-KNAW Collection), the Netherlands: *A. keratinophilus* (= *Chrysosporium keratinophilum*) (CBS 104.62), *Ch. tropicum* (CBS 171.62), and *Ch. pannicola* (CBS 116.63).

### Evaluation of the colony size and dispersion of *Aphanoascus keratinophilus* strains by rooks

Analyses of the number of nests in colonies were carried out between 15 and 30 April 2013 according to recommendations^54^. The colony sizes were as follows: Sielec—501 nests, Chojno Nowe—396 nests, Siennica—271 nests, Wola Uhruska—163 nests, Chełm (2 combined colonies) with 162 nests, and Wierzbica—59 nests. The results obtained by Luniak (^55^ point to the fact that one bird produces an average of 0.9 pellets/day, allowing estimation of the size of pellets produced by rook colonies. The pellets collected in the breeding period from March 15 to June 30, i.e., 107 days, were examined. Rooks usually forage 0.5–1.0 km from the breeding colony and devote a relatively long time toward brooding and brood protection^7^. It was estimated that adult birds cast all produced pellets within a 1 km radius from the colony centre, i.e., in an area of approximately 3.14 km^2^. The calculated production of pellets by adult birds during the entire breeding cycle in colonies allowed us to estimate the minimal dispersions of *A. keratinophilus* strains occurring in these colonies with the assumption that 1 pellet contains at least 1 strain. The total number of pellets produced in particular colonies was multiplied by the percentage of pellets, in which the presence of *A. keratinophilus* was confirmed by molecular analyses.

### Fungal strains used for molecular analysis

In this study, 99 strains of *Chrysosporium* sp*.* (Table 4) isolated from rook pellets were used for molecular identification. Among them, 90 strains of *A. keratinophilus* (Table 4) were used for molecular differentiation. The reference strains of *A. keratinophilus* (= *Ch. keratinophilum*), *Ch. tropicum,* and *Ch. pannicola* purchased from Westerdijk Fungal Biodiversity Institute (formerly CBS-KNAW Collection), the Netherlands were used.

**Table 4:** Fungal strains of *Chrysosporium* sp. isolated from rook pellets used in the present study.

Species (no. of strains)	Locality/no. of strains					
	Chełm	Chojno Nowe	Wola Uhruska	Sielec	Siennica	Wierzbica
*A. keratinophilus* (90)	50	13	15	3	6	3
*Ch. pannicola* (6)	3	0	0	0	3	0
*Ch. tropicum* (3)	0	0	0	0	3	0
Total no. of strains (99)	53	13	15	3	12	3

### DNA extraction

Total cellular DNA was extracted from a small amount of mycelium cultured on a Saboraud’s agar slant by a rapid mini-preparation method^56^. The mycelium was added to 700 μL of lysis buffer (400 mM Tris–HCl, 60 mM EDTA, 150 mM NaCl, 1% SDS) and incubated at 60 °C for 1 h. After the addition of 210 μL of 3 M sodium acetate, the homogenate was centrifuged at 12000 rpm for 15 min. The supernatant was successively extracted with phenol–chloroform-isoamyl alcohol (25:24:1). DNA was treated with RNase at a final concentration of 50 μg/mL for 20 min at 57 °C. Then, the samples were precipitated using 3 volumes of cold ethanol in the presence of 300 mM sodium acetate, and DNA was centrifuged for 10 min. The pellet was washed with 70% ethanol and air dried. DNA was dissolved in 30 μL TE buffer, and 1 μL of the resulting solution was used as a template in the following PCR**.**

### Molecular identification by PCR–RFLP

The ITS1, 5.8S, and ITS2 regions were amplified using the conserved primers ITS1 (5’-TCCGTAGTGGAACCTGCGG-3’) and ITS4 (5’ TCCTCCGCTTATTGATATGC-3’)^33^. Each PCR mixture (30 μL) contained 1 μL of genomic DNA, 1 μL of 50 pmol of each primer, 12 μL of distilled water and 15 μL of Taq PCR Master Mix Kit (Qiagen). Reaction mixtures were preheated to 95 °C for 15 min, and then 30 PCR cycles were performed under the following conditions: 95 °C for 1 min; 56 °C for 1 min; and 72 °C for 1 min. The thermal cycles were finalized by polymerization at 72 °C for 10 min^34^. Detection of PCR products was performed by electrophoresis in a 1% agarose gel stained with ethidium bromide and visualized by UV light. Ninety-nine PCR products of the analysed keratinophilic fungi and reference strains obtained using the ITS1 and ITS4 sets of primers were digested with the *Hin*fI (Fermentas) restriction enzyme at 37 °C for 2 h, according to the manufacturer’s instructions. Digested fragments were separated by electrophoresis in an 8% polyacrylamide gel, stained with ethidium bromide and visualized by UV light. Additionally, traditional identification was confirmed by sequencing selected PCR products (ITS1-5.8S rDNA-ITS4) of *A. keratinophilus* strains. The results obtained were compared with GenBank data.

### PCR-MP genotyping

The PCR-MP procedure was optimized according to the method described for dermatophytes^18^. DNA was digested with the *Bam*HI (10 U/µL) restriction enzyme (ThermoScientific). Digestion reactions were performed under uniform conditions: approximately 200 ng of the DNA sample was added to 2.5 µL of Buffer *Bam*HI (10 × concentrated, ThermoScientific) and 0.5 µL (5 U) of endonuclease in a total volume of 25 µL. After incubation at 37 °C for 3 h, the following ligation mix was prepared: 2 µL of two oligonucleotides (20 pmol of each) that formed an adapter, 2.5 µL of the ligation buffer [400 mM Tris–HCl pH 7.8, 100 mM MgCl_2,_ 100 mM dithiothreitol, 5 mM ATP; (ThermoScientific), and 0.1 µL of T4 DNA ligase (0.5U; ThermoScientific). The samples were then incubated at 22 °C overnight. PCR was carried out in a 25 µL reaction mixture containing 1 µL ligation solution, 2.5 µL 10 × PCR buffer *Shark* [200 mM Tris–HCl pH 8.8, 100 mM KCl, 100 mM (NH_4_)_2_SO_4_, 1% Triton X-100; DNA Gdańsk], 0.5 µL dNTP mix (200 µM each), 0.5 µL (1U) *Shark* polymerase (DNA Gdańsk) and 25 pmol MP-B primer (5’-CTC ACT CTC ACC AAC GTC GAC GAT CC-3’). The denaturation temperature was determined by specific optimization experiments with DNA of the reference strains and the number of *A. keratinophilus* isolates using a gradient thermal cycler (Thermal Cycler C1000™, BIORAD) within a gradient range of 80.0–84.0 °C. The PCRs were performed as follows: 7 min at 72 °C; initial denaturation for 90 s over a gradient of 80.0–84.0 °C, 24 cycles of denaturation for 1 min at a gradient of 80.0–84.0 °C, annealing and elongation step at 72 °C for 2 min 15 s, and final elongation at 72 °C for 5 min. PCRs for *A. keratinophilus* were performed as described above, using the established optimal denaturation temperature of 83 °C. All analyses were performed in triplicate, and PCR was performed using two different thermal cyclers: Thermal Cycler C1000™ (BIORAD) and Labcycler 48 Gradient (SensoQuest Biomedical Electronics). Electrophoresis of all PCR products was performed on 6% polyacrylamide gels.

### PCR-MP data analysis

Data analysis was conducted using BioNumerics software, Version 7.1. Strains with identical sizes and numbers of well-defined bands in the gels were considered genetically indistinguishable and were assigned to the same type. Strains with banding patterns that differed by up to three bands were considered closely related and were described as subtypes. Strains with banding patterns that differed by four or more bands were considered different types. The discriminatory power (D) of the PCR-MP typing, calculated for *A. keratinophilus,* was determined using Simpson's index diversity^57^:

$$D = 1 - \frac{1}{N(N - 1)}\sum\limits_{j = 1}^{S} {n_{j} (n_{j} - 1)}$$

where: N is the total number of strains of tested species, S is the total number of types (*j*), *n*_*j*_ is the number of strains within a specific type (*j*).

The (D) value range is from 0 to 1. Values close to 1 indicate a high discriminatory power of the analysed method.

### Estimation of PCR-MP genotype dispersal by rooks

Based on the molecular genotyping of *A. keratinophilus* strains isolated from rook pellets and data such as colony size, breeding duration, and daily pellet production of adult birds, the dispersion of PCR-MP genotypes was estimated. Due to the nature of the urban environment, the results for the two urban colonies of *Corvus frugilegus* in the city of Chełm (< 2 km) were combined (I. Kitowski—unpublished data).

### Statistical analysis

Statistical analysis was performed using the STATISTICA software system (version 12, StatSoft, Inc., Tulsa, OK, USA). The frequencies of the analysed strains of keratinophilic fungi were analysed using the 2 × 2 contingency Chi-square^58^.

## Supplementary Information

Supplementary Information.

## References

[1] Dynowska M, Meissner W, Pacyńska J (2013). Mallard duck (*Anas platyrhynchos*) as a potential link in the epidemiological chain mycoses originating from water reservoirs. Bull. Vet. Inst. Pulawy.

[2] Georgopoulou I, Tsiouris V (2008). The potential role of migratory birds in the transmission of zoonoses. Vet. Ital..

[3] Hubálek Z (2004). An annotated checklist of pathogenic microorganisms associated with migratory birds. J. Wildl. Dis..

[4] Korniłłowicz TKI (2009). Diversity of fungi in nests and pellets of Montagu’s harrier (*Circus pygargus*) from eastern Poland—Importance of chemical and ecological factors. Ecol. Chem. Eng..

[5] Korniłłowicz-Kowalska T, Kitowski I (2013). *Aspergillus fumigatus* and other thermophilic fungi in nests of wetland birds. Mycopathologia.

[6] Kiziewicz B. The occurrence fungi and zoosporic fungi-like organisms on feathers of birds Corvidae. in *Corvids of Poland* (ed. Jerzak, L.). 147–154. (Bogucki Wydawnictwo Naukowe Poznan, 2005).

[7] Kasprzykowski Z (2003). Habitat preferences of foraging Rooks *Corvus frugilegus* during the breeding period in the agricultural landscape of eastern Poland. Acta Ornithol..

[8] Czarnecka J, Kitowski I (2010). Seed dispersal by the rook *Corvus frugilegus* l. In agricultural landscape—Mechanisms and ecological importance. Polish J. Ecol..

[9] Czarnecka J (2013). Seed dispersal in urban green space—Does the rook *Corvus frugilegus* L. contribute to urban flora homogenization?. Urban For. Urban Green..

[10] Gromadzka J (1980). Food composition and food consumption of the Rook *Corvus frugilegus* in agrocoenoses in Poland. Acta Ornithol..

[11] Green AJ, Elmberg J, Lovas-Kiss Á (2019). Beyond scatter-hoarding and frugivory: European corvids as overlooked vectors for a broad range of plants. Front. Ecol. Evolut..

[12] Jędrzejewski, S., Majewska, A., Zduniak, P. & Graczyk, T. Parasites of Polish corvids—Knowledge and potential risk for human. in *Corvids of Poland* (eds. Jerzak, L., Kavanagh, B. P. & Trojanowski, P.). 137–145. (Bogucki Wydawnictwo Naukowe, 2005).

[13] Kiziewicz, B. The occurrenceof fungy and zoosporic fungus like organisms on feathers of birds Corvids. in *Corvids in Poland.* (eds. Jerzak, L., Kavanagh, B. P. & Trojanowski, P.). 147–154. (Bogucki Wydawnictwo Naukowe, 2005).

[14] Camin AM, Chabasse D, Guiguen C (1998). Keratinophilic fungi associated with starlings (*Sturnus vulgaris*) in Brittany, France. Mycopathologia.

[15] Hubálek Z (2000). Keratinophilic fungi associated with free-living mammals and birds. Biol. Dermatophytes Keratinophilic Fungi.

[16] Mandeel Q, Nardoni S, Mancianti F (2011). Keratinophilic fungi on feathers of common clinically healthy birds in Bahrain. Mycoses.

[17] Ciesielska A, Kawa A, Kanarek K, Soboń A, Szewczyk R (2021). Metabolomic analysis of *Trichophyton rubrum* and *Microsporum canis* during keratin degradation. Sci. Rep..

[18] Leibner-Ciszak, J., Dobrowolska, A., Krawczyk, B., Kaszuba, A. & Sta̧czek, P. Evaluation of a PCR melting profile method for intraspecies differentiation of *Trichophyton rubrum* and *Trichophyton interdigitale*. *J. Med. Microbiol.* **59**, 185–192 (2010). 10.1099/jmm.0.013458-0 19892858

[19] Ciesielska A, Oleksak B, Stączek P (2019). Reference genes for accurate evaluation of expression levels in *Trichophyton interdigitale* grown under different carbon sources, pH levels and phosphate levels. Sci. Rep..

[20] Calvo A, Vidal M, Guarro J (1984). Keratinophilic fungi from urban soils of Barcelona, Spain. Mycopathologia.

[21] R.S/, C. Taxonomy of the Onygenales: Arthrodermataceae, Gymnoasceae, Myxotrichaceae and Onygenaceae. *Mycotaxon* **24**, 1–216 (1985).

[22] Korniłłowicz-Kowalska T (1997). Studies on the decomposition of keratin wastes by saprotrophic microfungi. P. I. Criteria for evaluating keratinolytic activity. Acta Mycol..

[23] van Oorschot CAN (1980). A revision of *Chrysosporium* and allied genera. Stud. Mycol..

[24] Domsch KH, Gams WATH (1980). Compedium of Soil Fungi.

[25] Gan GG (2002). Non-sporulating Chrysosporium: An opportunistic fungal infection in a neutropenic patient. Med. J. Malaysia.

[26] de Hoog GS, Guarro J, Gene J (2001). Atlas of clinical fungi. Int. Microbiol.

[27] Manzano-Gayosso P (2008). Onychomycosis incidence in type 2 diabetes mellitus patients. Mycopathologia.

[28] Palma MAG, Espín LA, Pérez AF (2007). Invasine sinusal mycosis due to *Chrysosporium tropicum*. Acta Otorrinolaringol. Esp..

[29] Stillwell WT, Rubin BO (1984). Chrysosporium, a new causative agent in osteomycelitis. Clin. Orthopaed. Relat. Res..

[30] Gueho EVJGR (1985). A new human case of Anixiopsis stercomia mycosis: Discussion of its taxonomy and pathogenicity. Mycoses.

[31] Nieuwenhuis BPS, James TY (2016). The frequency of sex in fungi. Philos. Trans. R. Soc. B Biol. Sci..

[32] Neubauer G, Sikora ACT (2011). Monitoring populacji ptaków Polski w latach 2008–2009. Biuletyn Monitoringu Przyrody.

[33] Jackson CJ, Barton RC, Evans EGV (1999). Species identification and strain differentiation of dermatophyte fungi by analysis of ribosomal-DNA intergenic spacer regions. J. Clin. Microbiol..

[34] Mochizuki T (2003). Restriction fragment length polymorphism analysis of ribosomal DNA intergenic regions is useful for differentiating strains of *Trichophyton mentagrophytes*. J. Clin. Microbiol..

[35] Garg AP, Gandotra S, Mukerji KG, Pugh GJF (1985). Ecology of keratinophilic fungi. Proc. Plant Sci..

[36] Abulreesh HH, Goulder R, Scott GW (2007). Wild birds and human pathogens in the context of ringing and migration. Ringing Migr..

[37] Prinzinger R, Preßmar A, Schleucher E (1991). Body temperature in birds. Comp. Biochem. Physiol. Part A Physiol..

[38] Summerbell RC (2000). Form and function in the evolution of dermatophytes. Rev. Iberoam. Micol..

[39] Warwick A, Ferrieri P, Burke B, Blazar BR (1991). Presumptive invasive *Chrysosporium* infection in a bone marrow transplant recipient. Bone Marrow Transplant.

[40] Kitowski, I., Ciesielska, A., Korniłłowicz-Kowalska, T., Bohacz, J., & Świetlicki, M. *Estimation of Chrysosporium keratinophilum Dispersal by the Rook Corvus frugilegus in Chełm (East Poland) in Urban Fauna-Animal, Man, and the City—Interactions and Relationships*. (Indykiewicz, P. & Böhner, J. eds). 263–269. (Art Studio, 2014)

[41] Gopal KA, Kalaivani V, Anandan H (2020). Pulmonary infection by *Chrysosporium* species in a preexisting tuberculous cavity. Int. J. Appl. Basic Med. Res..

[42] Krawczyk B, Samet A, Leibner J, Śledzińska A, Kur J (2006). Evaluation of a PCR melting profile technique for bacterial strain differentiation. J. Clin. Microbiol..

[43] Ciesielska A (2014). Application of microsatellite-primed PCR (MSP-PCR) and PCR melting profile (PCR-MP) method for intraspecies differentiation of dermatophytes. Pol. J. Microbiol..

[44] Orłowski G, Czapulak A (2007). Different extinction risks of the breeding colonies of rooks *Corvus frugilegus* in rural and urban areas of SW Poland. Acta Ornithologica.

[45] Bohacz J, Korniłłowicz-Kowalska T (2012). Species diversity of keratinophilic fungi in various soil types. Cent. Eur. J. Biol..

[46] Papini R, Mancianti F, Grassotti G, Cardini G (1998). Survey of keratinophilic fungi isolated from city park soils of Pisa, Italy. Mycopathologia.

[47] Singh IKR (2010). Dermatophytes and related keratinophilic fungi in soil of parks and agricultural fields of Uttar Pradesh, India. Indian J. Dermatol..

[48] Gungnani HC, Sharma S, Gupta B (2012). Keratinophilic fungi recovered from feathers of different species of birds in St Kitts and Nevis. West Indian Med. J..

[49] Jadczyk P, J. Z. Wintering of rooks *Corvus frugilegus* in Poland. in *Corvids of Poland* (ed. Jerzak, L.). 541–556. (Bogucki Wydawnictwo Naukowe Poznan, 2005).

[50] Wilk, T., Chodkiewicz, T., Sikora, A., Chylarecki, P. & Kuczyński, L. *Red List of Polish Birds.* (OTOP, 2020).

[51] Oke, T.R. The heat island of the urban boundary layer: Characteristics, causes and effects. in *eWind Climate in Cities. NATO ASI Series E* (ed. JE, C.). 81–107. (Kluwer Academy, 1995).

[52] Vidal P, de Vinuesa M, Los A, Sánchez-Puelles JM, Guarro J (2000). Phylogeny of the anamorphic genus *Chrysosporium* and related taxa based on rDNA internal transcribed spacer sequences. Rev. Iberoam. Micol..

[53] Korniłłowicz T (1991). Studies on mycoflora colonizing raw keratin wastes in arable soil. Mycologica.

[54] Orłowski G, Kasprzykowski Z, Zawada Z, Kopij G (2009). Stomach content and grit ingestion by rook *Corvus frugilegus* nestlings. Ornis Fennica.

[55] Luniak M (1977). Consumption and digestion of food in the rook, *Corvus frugilegus*, in the condition of an aviary. Acta Ornithol..

[56] Liu D, Coloe S, Baird R, Pedersen J (2000). Rapid mini-preparation of fungal DNA for PCR [5]. J. Clin. Microbiol..

[57] Hunter PR, Gaston MA (1988). Numerical index of the discriminatory ability of typing systems: An application of Simpson’s index of diversity. J. Clin. Microbiol..

[58] Greenwell, J. R. Introduction to biostatistics, 2nd edn. By R. R. Sokal and F. J. Rohlf. pp. 363. F. H. Freeman and Co., 1987. £44.99 hardback. ISBN 0 7167 18057. *Exp. Physiol.* **80**, 681 (1995)

