# Supplementary material for: Dispersal of Aphanoascus keratinophilus by the rook Corvus frugilegus during breeding in East Poland
Source: Sci Rep. 2022 Feb 8;12:2142. doi: 10.1038/s41598-022-06227-2 (PMC8826369; doi:10.1038/s41598-022-06227-2)
Supplement: Supplementary file 1 — Supplementary Information. [file 41598_2022_6227_MOESM1_ESM.pdf]

SUPPLEMENTARY INFORMATIONS

Dispersal of *Aphanoascus keratinophilus* by the Rook *Corvus frugilegus* during breeding in East Poland

Ignacy Kitowski[1*], Teresa Korniłłowicz-Kowalska[2], Justyna Bohacz[2] and Anita Ciesielska[3*]

*Corresponding author: anita.ciesielska@biol.uni.lodz.pl

FIGURES:

Figure S1a,b Full-length gels concerning Figure 2

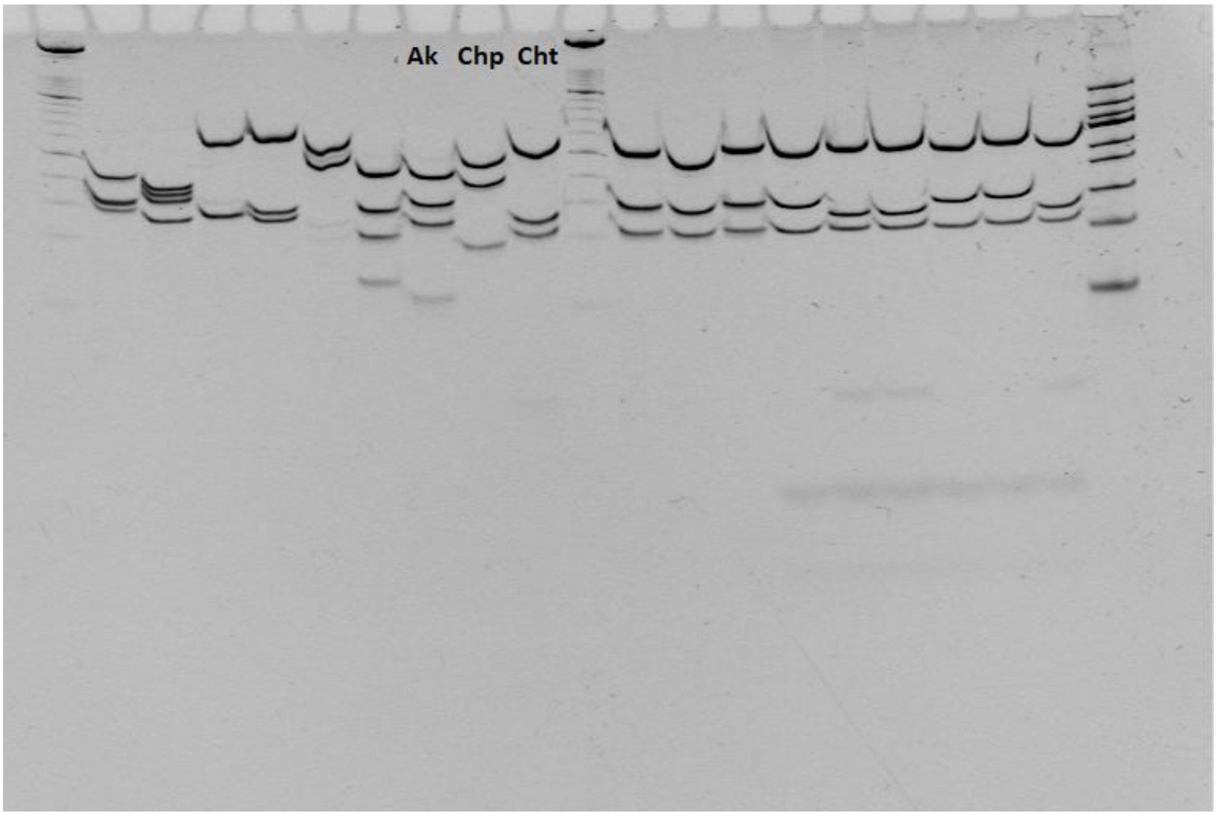

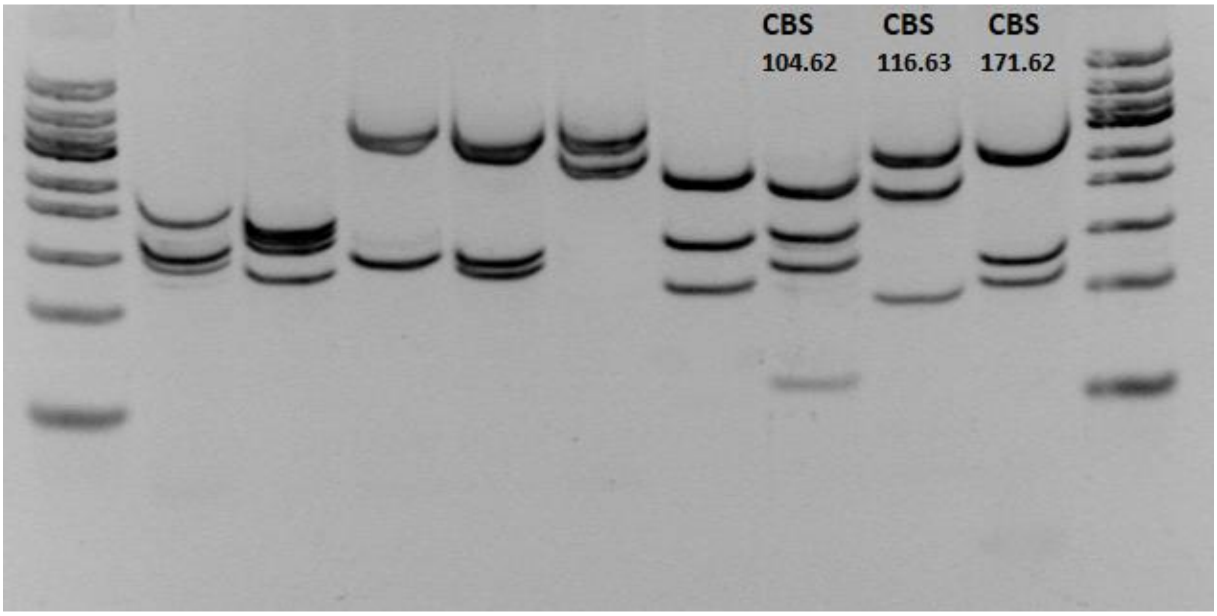
CBS
104.62
CBS
116.63
CBS
171.62

TABLE:

Table S1 Keratinophilic fungi isolated from the pellets of *Corvus frugilegus* used in this study

Name of strain of keratinolytic fungi isolated from Rook		Traditional identification	PCR-RFLP identification	Localization of rook's pellets (City)
1.	WŁ 119/3	*Chrysosporium sp.*	*Aphanoascus keratinophilus*	Chełm
2.	K 119/4	*Chrysosporium sp.*	*Aphanoascus keratinophilus*	Chełm
3.	K 121/1	*Chrysosporium sp.*	*Aphanoascus keratinophilus*	Chełm
4.	K 179/1	*Chrysosporium sp.*	*Aphanoascus keratinophilus*	Wierzbica
5.	K 179/3	*Chrysosporium sp.*	*Aphanoascus keratinophilus*	Wierzbica
6.	K 179/4	*Chrysosporium sp.*	*Aphanoascus keratinophilus*	Wierzbica
7.	K 191/1	*Chrysosporium sp.*	*Aphanoascus keratinophilus*	Chełm-hospital
8.	K 191/2	*Chrysosporium sp.*	*Aphanoascus keratinophilus*	Chełm
9.	K 213/1	*Chrysosporium sp.*	*Chrysosporium pannicola*	Siennica
10.	K 213/4	*Chrysosporium sp.*	*Chrysosporium pannicola*	Siennica
11.	K 121/2	*Chrysosporium tropicum*	*Aphanoascus keratinophilus*	Chełm
12.	K 121/3	*Chrysosporium tropicum*	*Aphanoascus keratinophilus*	Chełm
13.	WŁ 120/2	*Aphanoascus keratinophilus*	*Aphanoascus keratinophilus*	Chełm
14.	WŁ 120/3	*Aphanoascus keratinophilus*	*Aphanoascus keratinophilus*	Chełm
15.	WŁ 120/4	*Aphanoascus keratinophilus*	*Aphanoascus keratinophilus*	Chełm
16.	WŁ 121/3	*Aphanoascus keratinophilus*	*Aphanoascus keratinophilus*	Chełm
17.	WŁ 121/4	*Aphanoascus keratinophilus*	*Aphanoascus keratinophilus*	Chełm
18.	WŁ 122/1	*Aphanoascus keratinophilus*	*Aphanoascus keratinophilus*	Chełm
19.	WŁ 123/2	*Aphanoascus keratinophilus*	*Aphanoascus keratinophilus*	Chełm
20.	WŁ 124/1	*Aphanoascus keratinophilus*	*Aphanoascus keratinophilus*	Chełm
21.	WŁ 124/2	*Aphanoascus keratinophilus*	*Aphanoascus keratinophilus*	Chełm
22.	WŁ 124/3	*Aphanoascus keratinophilus*	*Aphanoascus keratinophilus*	Chełm
23.	WŁ 129/1	*Aphanoascus keratinophilus*	*Aphanoascus keratinophilus*	Chełm
24.	WŁ 129/2	*Aphanoascus keratinophilus*	*Aphanoascus keratinophilus*	Chełm
25.	WŁ 129/3	*Aphanoascus keratinophilus*	*Aphanoascus keratinophilus*	Chełm
26.	WŁ 132/1	*Aphanoascus keratinophilus*	*Aphanoascus keratinophilus*	Chełm
27.	WŁ 132/2	*Aphanoascus keratinophilus*	*Aphanoascus keratinophilus*	Chełm
28.	WŁ 133/1	*Aphanoascus keratinophilus*	*Aphanoascus keratinophilus*	Chełm
29.	WŁ 133/2	*Aphanoascus keratinophilus*	*Aphanoascus keratinophilus*	Chełm
30.	WŁ 133/4	*Aphanoascus keratinophilus*	*Aphanoascus keratinophilus*	Chełm
31.	WŁ 134/4	*Aphanoascus keratinophilus*	*Aphanoascus keratinophilus*	Chełm
32.	WŁ 172/2	*Aphanoascus keratinophilus*	*Aphanoascus keratinophilus*	Wola Uhruska
33.	WŁ 172/4	*Aphanoascus keratinophilus*	*Aphanoascus keratinophilus*	Wola Uhruska
34.	WŁ 190/1	*Aphanoascus keratinophilus*	*Aphanoascus keratinophilus*	Chełm-hospital
35.	WŁ 190/2	*Aphanoascus keratinophilus*	*Aphanoascus keratinophilus*	Chełm-hospital
36.	WŁ 190/3	*Aphanoascus keratinophilus*	*Aphanoascus keratinophilus*	Chełm-hospital
37.	K 119/2	*Aphanoascus keratinophilus*	*Aphanoascus keratinophilus*	Chełm
38.	K 119/3	*Aphanoascus keratinophilus*	*Aphanoascus keratinophilus*	Chełm
39.	K 120/1	*Aphanoascus keratinophilus*	*Aphanoascus keratinophilus*	Chełm
40.	K 120/2	*Aphanoascus keratinophilus*	*Aphanoascus keratinophilus*	Chełm
41.	K 120/3	*Aphanoascus keratinophilus*	*Aphanoascus keratinophilus*	Chełm
42.	K 124/1	*Aphanoascus keratinophilus*	*Aphanoascus keratinophilus*	Chełm
43.	K 124/2	*Aphanoascus keratinophilus*	*Aphanoascus keratinophilus*	Chełm
44.	K 132/1	*Aphanoascus keratinophilus*	*Aphanoascus keratinophilus*	Chełm
45.	K 133/1	*Aphanoascus keratinophilus*	*Aphanoascus keratinophilus*	Chełm
46.	K 133/2	*Aphanoascus keratinophilus*	*Aphanoascus keratinophilus*	Chełm
47.	K 134/2	*Aphanoascus keratinophilus*	*Aphanoascus keratinophilus*	Chełm
48.	K 134/3	*Aphanoascus keratinophilus*	*Aphanoascus keratinophilus*	Chełm
49.	WŁ 175/3	*Aphanoascus keratinophilus*	*Aphanoascus keratinophilus*	Wola Uhruska
50.	WŁ 176/1	*Aphanoascus keratinophilus*	*Aphanoascus keratinophilus*	Wola Uhruska
51.	WŁ 176/2	*Aphanoascus keratinophilus*	*Aphanoascus keratinophilus*	Wola Uhruska
52.	WŁ 207/1	*Aphanoascus keratinophilus*	*Aphanoascus keratinophilus*	Siennica
53.	WŁ 207/2	*Aphanoascus keratinophilus*	*Aphanoascus keratinophilus*	Siennica

54. Wł 207/3	*Aphanoascus keratinophilus*	*Aphanoascus keratinophilus*	Siennica
55. Wł 220/2	*Aphanoascus keratinophilus*	*Aphanoascus keratinophilus*	Sielec
56. Wł 231/1	*Aphanoascus keratinophilus*	*Aphanoascus keratinophilus*	Chojno Nowe
57. Wł 231/3	*Aphanoascus keratinophilus*	*Aphanoascus keratinophilus*	Chojno Nowe
58. Wł 231/4	*Aphanoascus keratinophilus*	*Aphanoascus keratinophilus*	Chojno Nowe
59. Wł 232/1	*Aphanoascus keratinophilus*	*Aphanoascus keratinophilus*	Chojno Nowe
60. Wł 232/3	*Aphanoascus keratinophilus*	*Aphanoascus keratinophilus*	Chojno Nowe
61. Wł 233/1	*Aphanoascus keratinophilus*	*Aphanoascus keratinophilus*	Chojno Nowe
62. Wł 233/2	*Aphanoascus keratinophilus*	*Aphanoascus keratinophilus*	Chojno Nowe
63. Wł 233/4	*Aphanoascus keratinophilus*	*Aphanoascus keratinophilus*	Chojno Nowe
64. Wł 236/1	*Aphanoascus keratinophilus*	*Aphanoascus keratinophilus*	Chojno Nowe
65. K 176/4	*Aphanoascus keratinophilus*	*Aphanoascus keratinophilus*	Wola Uhruska
66. K 177/1	*Aphanoascus keratinophilus*	*Aphanoascus keratinophilus*	Wola Uhruska
67. K 177/2	*Aphanoascus keratinophilus*	*Aphanoascus keratinophilus*	Wola Uhruska
68. K 207/1	*Aphanoascus keratinophilus*	*Aphanoascus keratinophilus*	Siennica
69. K 207/3	*Aphanoascus keratinophilus*	*Aphanoascus keratinophilus*	Siennica
70. K 207/4	*Aphanoascus keratinophilus*	*Aphanoascus keratinophilus*	Siennica
71. K 236/1	*Aphanoascus keratinophilus*	*Aphanoascus keratinophilus*	Chojno Nowe
72. K 236/2	*Aphanoascus keratinophilus*	*Aphanoascus keratinophilus*	Chojno Nowe
73. K 236/3	*Aphanoascus keratinophilus*	*Aphanoascus keratinophilus*	Chojno Nowe
74. K 129/1	*Aphanoascus keratinophilus*	*Chrysosporium pannicola*	Chełm
75. K 129/4	*Aphanoascus keratinophilus*	*Chrysosporium pannicola*	Chełm
76. K 134/1	*Aphanoascus keratinophilus*	*Chrysosporium pannicola*	Chełm
77. Wł 123/1	*Aphanoascus fulvescens*	*Aphanoascus keratinophilus*	Chełm
78. Wł 123/3	*Aphanoascus fulvescens*	*Aphanoascus keratinophilus*	Chełm
79. Wł 172/5	*Aphanoascus fulvescens*	*Aphanoascus keratinophilus*	Wola Uhruska
80. K 123/1	*Aphanoascus fulvescens*	*Aphanoascus keratinophilus*	Chełm
81. K 123/2	*Aphanoascus fulvescens*	*Aphanoascus keratinophilus*	Chełm
82. K 123/3	*Aphanoascus fulvescens*	*Aphanoascus keratinophilus*	Chełm
83. K 133/3	*Aphanoascus fulvescens*	*Aphanoascus keratinophilus*	Chełm
84. Wł 175/1	*Aphanoascus fulvescens*	*Aphanoascus keratinophilus*	Wola Uhruska
85. Wł 175/2	*Aphanoascus fulvescens*	*Aphanoascus keratinophilus*	Wola Uhruska
86. Wł 176/3	*Aphanoascus fulvescens*	*Aphanoascus keratinophilus*	Wola Uhruska
87. Wł 220/1	*Aphanoascus fulvescens*	*Aphanoascus keratinophilus*	Sielec
88. Wł 220/3	*Aphanoascus fulvescens*	*Aphanoascus keratinophilus*	Sielec
89. Wł 232/2	*Aphanoascus fulvescens*	*Aphanoascus keratinophilus*	Chojno Nowe
90. K 176/1	*Aphanoascus fulvescens*	*Aphanoascus keratinophilus*	Wola Uhruska
91. K 176/2	*Aphanoascus fulvescens*	*Aphanoascus keratinophilus*	Wola Uhruska
92. K 177/3	*Aphanoascus fulvescens*	*Aphanoascus keratinophilus*	Wola Uhruska
93. K 204/1	*Aphanoascus fulvescens*	*Aphanoascus keratinophilus*	Chełm-hospital
94. K 204/3	*Aphanoascus fulvescens*	*Aphanoascus keratinophilus*	Chełm-hospital
95. K 204/4	*Aphanoascus fulvescens*	*Aphanoascus keratinophilus*	Chełm-hospital
96. Wł 210/3	*Aphanoascus fulvescens*	*Chrysosporium pannicola*	Siennica
97. K 208/1	*Aphanoascus fulvescens*	*Chrysosporium tropicum*	Siennica
98. K 208/2	*Aphanoascus fulvescens*	*Chrysosporium tropicum*	Siennica
99. K 208/3	*Aphanoascus fulvescens*	*Chrysosporium tropicum*	Siennica

Isolates which showed differences between morphological and molecular identification are marked in color.

Results

DNA sequencing analysis

BLAST query revealed that the obtained sequences of the three misidentified strains of *A. keratinophilus* showed 98% homology with the sequence of *Chrysosporium pannicola* (=*Ch. evolceanui*) (Accession no. AJ005368.1) (in 3 cases). On the other hand, 2 strains misidentified as *Ch. tropicum* BLAST query revealed 98% homology with the sequence of *Chrysosporium keratinophilum* (=*A. keratinophilus*) strain 55159 (Accession no. AB361655.1). In the case of 10 strains identified by the traditional method as *Chrysosporium* sp. based on sequencing analysis revealed 98% homology with the sequence of *Chrysosporium keratinophilum* (=*A. keratinophilus*) (Accession no. AB361655.1) (in 8 cases) and 96% homology to sequence of *Chrysosporium pannicola* (=*Ch. evolceanui*) (Accession no. AJ005368.1) (in 2 cases). The twenty misidentified strains of A. *fulvescens* showed 96% homology with the sequence of *Chrysosporium keratinophilum* (=*A. keratinophilus*) strain 55159 (Accession no. AB361655.1) (in 19 cases) and 95% homology to the sequence of *Chrysosporium tropicum* isolate AW31L (Accession no. KP735246.1) (in 1 case). 3 strains misidentified as *A. fulvescens* based on sequencing analysis revealed 95% homology with *Chrysosporium tropicum* isolate AW31L (Accession no. KP735246.1) (*data not shown*).
